# Vitamin C promotes decitabine or azacytidine induced DNA hydroxymethylation and subsequent reactivation of the epigenetically silenced tumour suppressor *CDKN1A* in colon cancer cells

**DOI:** 10.18632/oncotarget.25999

**Published:** 2018-08-28

**Authors:** Christian Gerecke, Fabian Schumacher, Alexander Edlich, Alexandra Wetzel, Guy Yealland, Lena Katharina Neubert, Bettina Scholtka, Thomas Homann, Burkhard Kleuser

**Affiliations:** ^1^ Institute of Nutritional Science, Department of Nutritional Toxicology, University of Potsdam, Nuthetal, Germany; ^2^ Department of Molecular Biology, University of Duisburg-Essen, Essen, Germany; ^3^ NutriAct – Competence Cluster Nutrition Research Berlin-Potsdam, Germany

**Keywords:** vitamin C, tumor suppressors, epigenetics, TET, DNA hydroxymethylation

## Abstract

Epigenetic silencing of tumour suppressor genes is a key hallmark of colorectal carcinogenesis. Despite this, the therapeutic potential of epigenetic agents capable of reactivating these silenced genes remains relatively unexplored. Evidence has shown the dietary antioxidant vitamin C (ascorbate) acts as an inducer of the ten-eleven translocation (TET) dioxygenases, an enzyme family that catalyses a recently described mechanism of DNA demethylation linked to gene re-expression. In this study, we set out to determine whether vitamin C can enhance the known anti-neoplastic actions of the DNA-demethylating agents decitabine (DAC) and azacytidine (AZA) in colorectal cancer cells. Administration of vitamin C alone significantly enhanced global levels of 5-hydroxymethyl-2’-deoxycytidine (5-hmdC), without altering 5-methyl-2’-deoxycytidine (5-mdC), as would be expected upon the activation of TET dioxygenases. Concomitant treatment of vitamin C with either AZA or DAC resulted in an unexpectedly high increase of global 5-hmdC levels, one that administration of any these compounds alone could not achieve. Notably, this was also accompanied by increased expression of the tumour suppressor *p21 (CDKN1A),* and a significant increase in apoptotic cell induction. Our *in vitro* data leads us to hypothesize that the reactivation of genes in colorectal cancer cells by AZA or DAC can be improved when the 5-hmdC levels are simultaneously increased by the TET activator vitamin C. The dual administration of demethylating agents and vitamin C to colorectal cancer patients, a demographic in which vitamin C deficiencies are common, may improve responses to epigenetic therapies.

## INTRODUCTION

Colorectal cancer is one of the most common neoplastic diseases of the western world [1]. Carcinogenesis of the colon and other tissue is a multi-levelled process characterised by the accumulation of numerous mutations and epigenetic aberrations [2, 3]. In addition to characteristic mutations in tumour suppressors such as *APC (adenomatous polyposis coli)* and genes of the *Wnt (wingless/lnt1)* signalling pathway [4], epigenetic aberrations are known to have an important impact on the neoplastic progression of the colonic epithelia [5–11]. Aberrant, loci-specific DNA hypermethylation – one of the best-studied epigenetic hallmarks of cancer – can lead to silencing of tumour suppressors that control, e.g. cell cycle and DNA repair processes [11, 12].

Initially, DNA methyltransferase (DNMT)-mediated DNA methylation was considered a non-reversible modification in mammals. It has since become clear, however, that the group of TET (ten-eleven translocation) dioxygenases are capable of oxidizing methylated 2’-deoxycytidines (5-methyl-2’-deoxycytidine; 5-mdC) to hydroxymethylated 2’-deoxycytidines (5-hydroxymethyl-2’-deoxycytidine, 5-hmdC) [13], a known mechanism of active DNA demethylation. TETs can further oxidize 5-hmdC to 5-formyl-2’-deoxycytidine (5-fdC) and 5-carboxy-2’-deoxycytidine (5-cadC), which are eventually replaced by unmodified 2’-deoxycytidines as a result of thymine-DNA glycosylase (TDG) mediated base excision repair [14–17]. This cascade of 5-mdC oxidation by TETs might represent the active demethylation mechanism leading to a genes transcriptional reactivation.

In addition to its role as a demethylation intermediate, 5-hmdC has unique epigenetic properties that result in specific gene expression profiles [18, 19]. Indeed, a genome-wide decrease in 5-hmdC- levels is considered an epigenetic hallmark in many cancers [20, 21] and several studies have highlighted the diagnostic and prognostic value of this mechanism [22]. From a therapeutic standpoint, there are a limited number of known TET enzyme activators capable of inducing significant increases to genome-wide 5-hmdC levels. Vitamin C (ascorbate) is one of the best-known substrates for TET enzymes. Unlike other mammals, humans are incapable of synthesising ascorbate *de novo*, making it an essential dietary requirement. It enters the cell by the sodium-dependent vitamin C transporter SVCT1, as expressed in intestinal and renal epithelial cells, or the ubiquitously expressed SVCT2. In human blood plasma, ∼50 μM ascorbate is considered optimal, < 11.4 μM is defined as a deficiency. Depending on the tissue, 1-10 mM ascorbate is found within cells [23]. It serves as an antioxidant, a radical scavenger, and an essential regenerative cofactor for Fe^2+^ and 2-oxoglutarate (2OG)-dependent dioxygenases (e.g. prolyl-4-hydroxylase [P4H], TET). In the latter, ascorbate reduces the inactive oxidised form of Fe^3+^ to the catalytically active Fe^2+^ in the active centre [24, 25]. Though the mode of ascorbate-induced TET dioxygenases activation is still controversial [26], it is clearly capable of re-establishing normal 5-hmdC-levels in cancer cells [21].

Many epigenetically potent compounds have been identified as potential cancer therapeutics that could play an important role in the management of neoplasias. Currently, two DNMT inhibitors – azanucleosides – have been approved by the US Food and Drug Administration (FDA) and the European Medicines Agency (EMA) for the treatment of certain forms of acute myeloid leukemia (AML) and myelodysplastic syndromes (MDS): 5-aza-cytidine (AZA; Vidaza^®^, Celgene, New Jersey, USA) [27–29] and 5-aza-2’-deoxycytidine (DAC; Dacogen^®^, Astex, Cambridge, UK) [30, 31]. As recent clinical trials suggest that azacytidine has wide therapeutic application in AML, it has been approved by the FDA for AML with 20 – 30% blasts in the bone marrow only, the EMA approved it for AML with 30% bone marrow blasts or higher [32]. Though both cytosine analogues were originally developed as cytostatic agents for leukaemia chemotherapy, their epigenetic properties have since been revealed to play an important role in their anti-cancer activity [31, 33–38]. Both act as DNMT inhibitors, DAC in particular, an action used for the treatment of myeloid malignancies such as AML. Indeed, both drugs show high efficiency against haematological cancers (MDS, AML) [39, 40]. Though AZA and DAC were once viewed as mechanistically similar working compounds, major differences in the mode of action by which they demethylate the cellular epigenome have since been revealed. During mitosis, DAC is exclusively incorporated into the newly synthesised DNA strand instead of 2’-deoxycytidine after cell division. Upon binding DAC, DNMT1 is inhibited and subsequently degraded by proteolysis. In contrast, 80-90% of AZA is incorporated into RNA and leading to mRNA and protein metabolism disruption and inhibition of malignant proliferation [38]. In addition, 10-15% of AZA is metabolised into DAC and can be introduced into DNA, as well [41, 42].

Although hypomethylating actions can be observed at concentrations as low as 30 nM DAC and 300 nM AZA, *in vitro* higher concentrations (3-10 μM DAC or AZA) prove to be more cytotoxic with no improvement to hypomethylation. In this regard, it is notable that such levels (3-11 μM AZA; 0.3–1.6 μM DAC) can be found in human plasma following normal administrations to cancer patients [43–45]. Higher concentrations of DAC or AZA (3-10 μM) do not lead to improved hypomethylating actions but more to cytotoxic effects which can also be found in human plasma (3-11 μM AZA) and (0.3–1.6 μM DAC) [43].

Several studies have shown that epigenetic therapies can induce re-expression of aberrantly silenced genes. An important example is the silencing of the potent tumour suppressors *p16 (CDKN2A)*, *p21 (CDKN1A)* and *p27 (CDKN1B)* [46]. Such loss of functions allow cells to bypass the signals for cell cycle control, apoptosis induction and senescence. Accordingly, the epigenetic therapy and reactivation by the action of demethylating substances, like DAC and AZA, led to a better prognosis of cancer patients [47–49].

Although DAC and AZA appear to be beneficial in combination with chemotherapy against solid tumours [50, 51], treatment with the demethylating agents alone is less effective. As already mentioned, DAC can only be used for epigenetic modulation at concentrations less than 3-11 μM, cytotoxicity becoming a prominent issue at higher doses [52, 53]. A multitude of new compounds for the epigenetic treatment of solid tumours are presently in clinical trials [38]. Innovative combinations of epigenetically active substances have been shown to improve therapeutic actions against solid tumours. However, the combination of DNMT inhibitors and compounds capable of reactivating DNA hydroxymethylation, such as vitamin C (ascorbate) [25, 54], is yet to be investigated as a novel therapeutic avenue in colorectal neoplasia.

This newly discovered function of vitamin C prompted us to determine whether additive or synergistic effects can be achieved when sub-cytotoxic concentrations of the DNMT inhibitors DAC and AZA are used in combination with ascorbate. Investigations were conducted in HCT116 cells, a commonly used colorectal cancer cell line, to assess whether such an epigenetic therapy has therapeutic application in colorectal cancers. Herein, we demonstrate the positive combinatorial effect ascorbate has upon the demethylating agents AZA and DAC, resulting in higher 5-hmdC-levels and increased expression of the tumour suppressor levels of *CDKN1A*.

## RESULTS

### Cell viability following exposure to DAC, AZA and vitamin C

**Figure 1:**
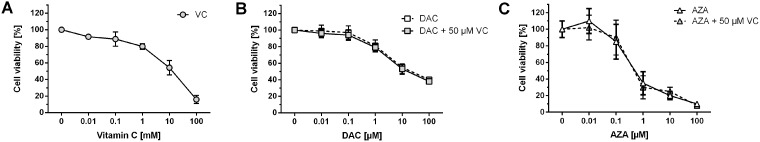
Effect of DAC, AZA and vitamin C on cell viability HCT116 cells were exposed to the indicated concentrations of vitamin C **(A)**, DAC **(B)** or AZA **(C)** alone or in combination with 50 μM vitamin C (grey boxes, DAC + VC – B; AZA + VC – C) for 72 h, and cell viabilities assessed by MTT assay. Data are expressed as a percentage of the untreated control, viable cell levels < 75% were taken to indicate cytotoxic induction (error bars = SD; n=3).

First, MTT (3-(4, 5-dimethylthiazol-2-yl)-2, 5-diphenyltetrazolium bromide) reduction assay was conducted to detect potential detrimental effects to the metabolic activity of HCT116 cells following 72 h treatment periods. Vitamin C was well tolerated up to 1 mM, though notable cytotoxicity was evident past this (Figure 1A). Well in line with previous studies [55], decreased cell viability was observed after exposure to 1 μM DAC or greater (Figure 1B), as illustrated by the white boxes with increasing DAC concentrations. However, after combined incubation of DAC and 50 μM vitamin C no additional cytotoxic effects were observed, as seen in Figure 1B. Exposure to AZA led to even greater reductions in cell viability at concentrations of 1 μM and above (Figure 1C). No additional toxic effects on cell viability were observed after exposure to indicated AZA concentrations in combination with 50 μM vitamin C (Figure 1C).

### Impact of AZA, DAC and vitamin C on global DNA methylation and ALU expression

**Figure 2:**
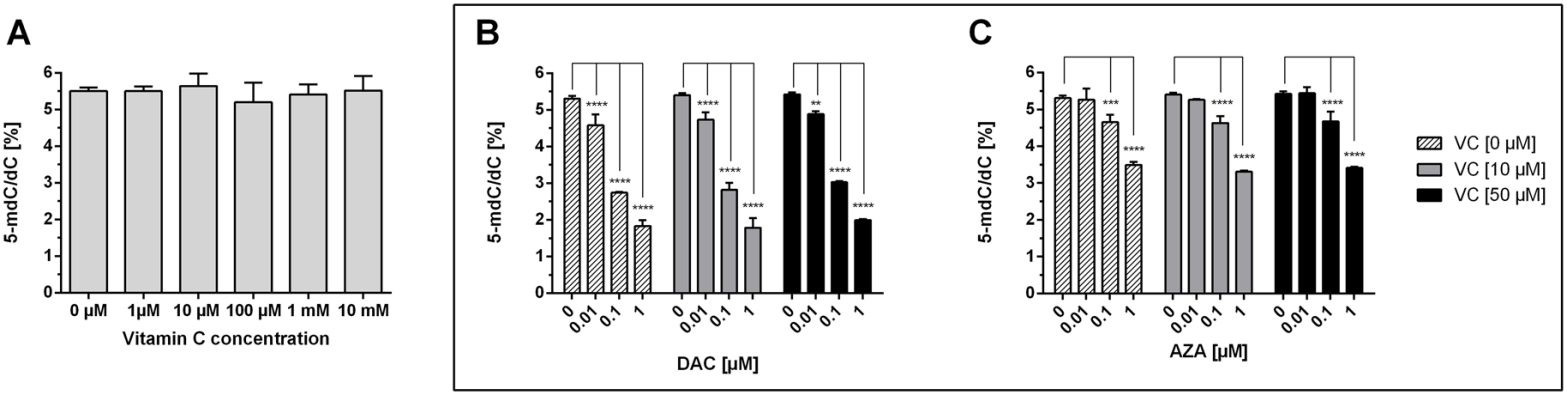
Impact of DAC, AZA and vitamin C on genomic 5-mdC/dC levels in human colon cancer cells HCT116 cells were exposed to increasing concentrations of vitamin C **(A)** for 72h. In addition, DAC **(B)** and AZA **(C)**, alone (striped bars) or in combination with 10 μM vitamin C (grey bars) and 50 μM vitamin C (black bars), were exposed to the HCT116 colon cancer cells for 72 h. The genomic levels of 5-mdC/dC were then assessed by LC-MS/MS. Statistical significance was calculated using 2-way ANOVA and Tukey post-test (^****^p < 0.001 – significant results; error bars = SD; n=3).

Next, we aimed to determine the genome-wide 5-mdC levels of colon cancer cells treated with vitamin C and DAC or AZA over 72 h, by isotope-dilution LC-MS/MS (Figure 2). Treatment with increasing concentrations of vitamin C alone did not lead to significant changes in the 5-mdC/dC-levels in the HCT116 cells, as illustrated by the grey bars (Figure 2A). In contrast, exposure to DAC significantly decreased the 5-mdC levels in a concentration-dependent manner; 5.5% to 2% [5-mdC/dC] from untreated to 1 μM DAC treated HCT116 cells, as shown by Figure 2B. Concentrations of DAC in 0.1 μM (p < 0.05) and 1 μM (p < 0.01) led to a significant decrease of 5-mdC/dC levels compared to the untreated control. Higher DAC doses did not further decrease 5-mdC/dC levels (data not shown). The combined treatment of HCT116 with 10 μM and 50 μM vitamin C did not alter the demethylating effect of DAC (Figure 2B). Incubation with increasing concentrations of AZA produced similar though weaker reductions; 5.5% to 3% [5-mdC/dC] from untreated to 1 μM AZA-treated cells (Figure 2C, black bars). Notably, only cytotoxic concentrations of AZA were capable of effecting large reductions to 5-mdC/dC levels in HCT116 cells not significantly. However, 0.1 μM AZA, as a non-toxic concentration, reduced the 5-mdC/dC levels in HCT116 cells not significantly. Treatment with 10 μM (bright grey bars) or 50 μM (dark grey bars) vitamin C and AZA (Figure 2C) had no apparent effect on genome-wide 5-mdC-levels. Accordingly, although treatment with DAC or AZA induced the expected dose dependent-decreases in 5-mdC levels, co-treatment with vitamin had no further influence on this effect (Figure 2B and 2C).

**Figure 3:**
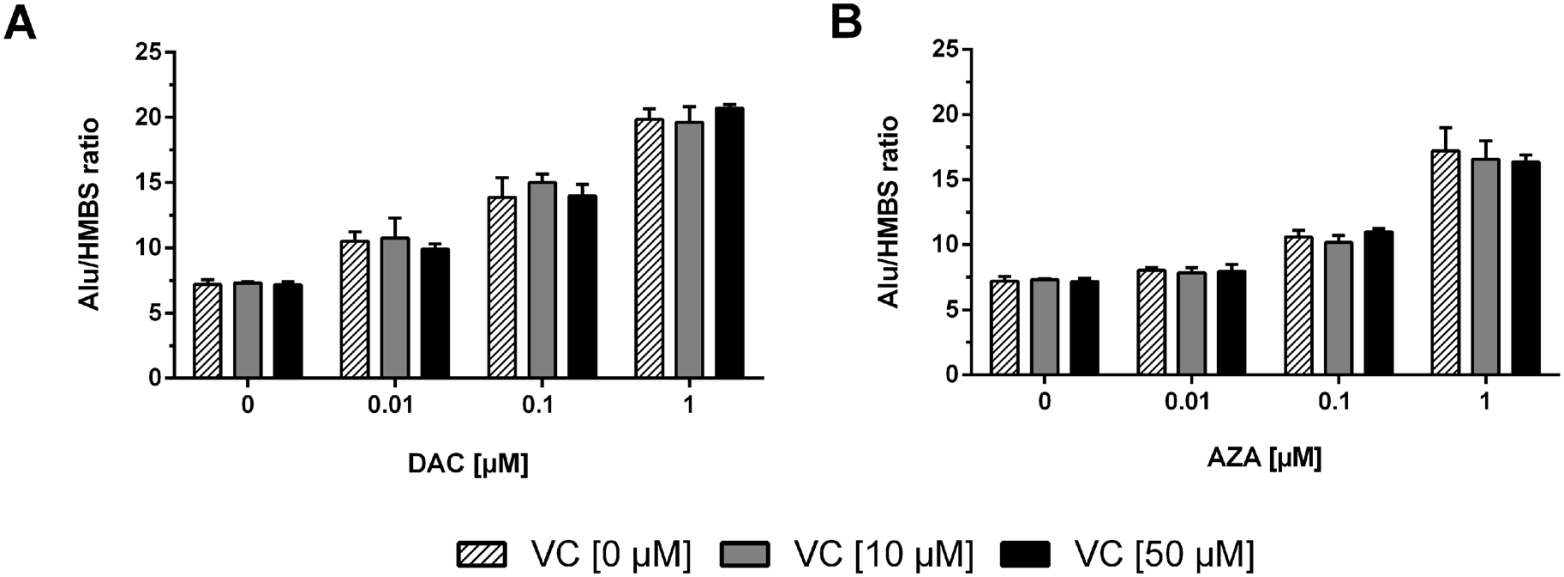
Reactivation of *ALU* transposable elements as control of epigenetic treatment and DNMT inhibition The mRNA levels of the *ALU1* transposon were measured after 72 h incubation with DAC **(A)** or AZA **(B)** alone or in combination with 10 μM (grey bars) or 50 μM (black bars) vitamin C. The relative quantitation was calculated by 2^-ΔΔCT^ method. *HMBS* served as control. (Columns: mean. Error bars: SD, n=3).

Genome-wide demethylation induced by DAC or AZA should lead to the re-expression of silenced transposable elements like *ALU* (*athrobacter luteus*). *ALU* elements can serve as effective markers for methylation and demethylation owing to their high abundance of CpG-sites and the high methylation levels found in their promoter region; these contribute up to 30% of genome-wide methylated DNA [56]. Concordant to the genome-wide loss of DNA methylation induced, we found DAC induced significant induction of *ALU* in a concentration-dependent manner (two-way ANOVA, p < 0.0001) that the addition of vitamin C did not alter (Figure 3A). Similar, though weaker dose-dependent reactivation of *ALU* expression was noted following AZA treatment (two-way ANOVA, p < 0.0001) (Figure 3B). Again, the addition of vitamin C at concentrations as high as 50 μM had no significant impact.

### Impact of AZA, DAC and vitamin C on global DNA hydroxymethylation

**Figure 4:**
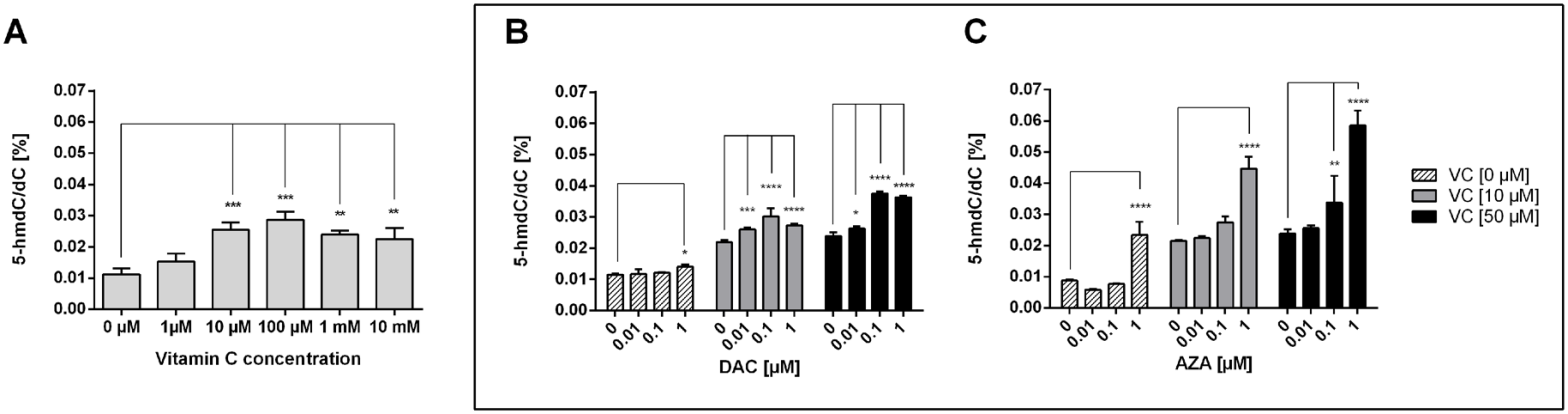
Impact of DAC, AZA and vitamin C on genomic 5-hmdC/dC levels in human colon cancer cells HCT116 cells were exposed to increasing concentrations of vitamin C **(A)** for 72 h. In addition, DAC **(B)** and AZA **(C)**, alone or in combination with vitamin C (10 μM and 50 μM), were exposed to the colon cancer cells for 72 h. The genomic levels of 5-hmdC/dC were then assessed by LC-MS/MS. Statistical significance was calculated using 2-way ANOVA and Tukey post-test (^*^p < 0.05, ^**^p < 0.01, ^***^ < 0.001, ^****^ < 0.0001– significant results for treatment groups. Error bars = SD; n=3).

As vitamin C is a potent activator of the TET enzymes and leads to an increase of genome-wide 5-hmdC/dC levels, we aimed to analyse the genome-wide DNA hydroxymethylation in vitamin C and DAC or AZA-treated HCT116 cells (Figure 4). Well in line with previous work, vitamin C treated HCT116 cells showed increased 5-hmdC levels in a dose-dependent manner (Figure 4A) [50]. At 100 μM, vitamin C was able to increase 5-hmdC/dC levels 2.55 fold (p < 0.001), although no further increases were seen above this. Interestingly, although DAC caused only negligible 5-hmdC/dC increases when applied alone, in combination with vitamin C its efficacy was greatly enhanced (Figure 4B). For instance, where 0.1 μM DAC alone increases 5-hmdC/dC levels 1.05 fold relative to untreated cells (two way ANOVA with Tukey post analysis for multiple comparisons, hereafter referred to as ‘Tukey’: p > 0.05), a 1.38 fold and a 1.57 fold increase relative to matched DAC only treated cells was seen in the presence of 10 and 50 μM vitamin C respectively (Tukey: p < 0.0001 in both instances). Although this effect saturated between 0.1 and 1 μM DAC within a given vitamin C concentration, it is possible that higher vitamin C concentrations could achieve even higher 5-hmdC/dC levels. Notably, greater 5-hmdC/dC levels were achievable with DAC/vitamin C combinatorial treatments than with even the most effective vitamin C concentration alone. A similar scenario is also seen when AZA is applied in combination with vitamin C. Although 1 μM AZA alone was capable of inducing a 2.69 fold increase in 5-hmdC/dC levels (p < 0.0001), when combined with 50 μM vitamin C, 0.1 μM AZA effected a 1.42 fold increase (p < 0.01), the only non-toxic AZA concentration to induce a significant change to 5-hmdC/dC levels (Figure 4). It is again notable that combinatorial AZA/vitamin C treatments were capable of inducing notably higher genome-wide 5-hdmC/dC levels than either alone.

### Expression of *TETs* and *DNMTs* in colon cancer cells

**Figure 5:**
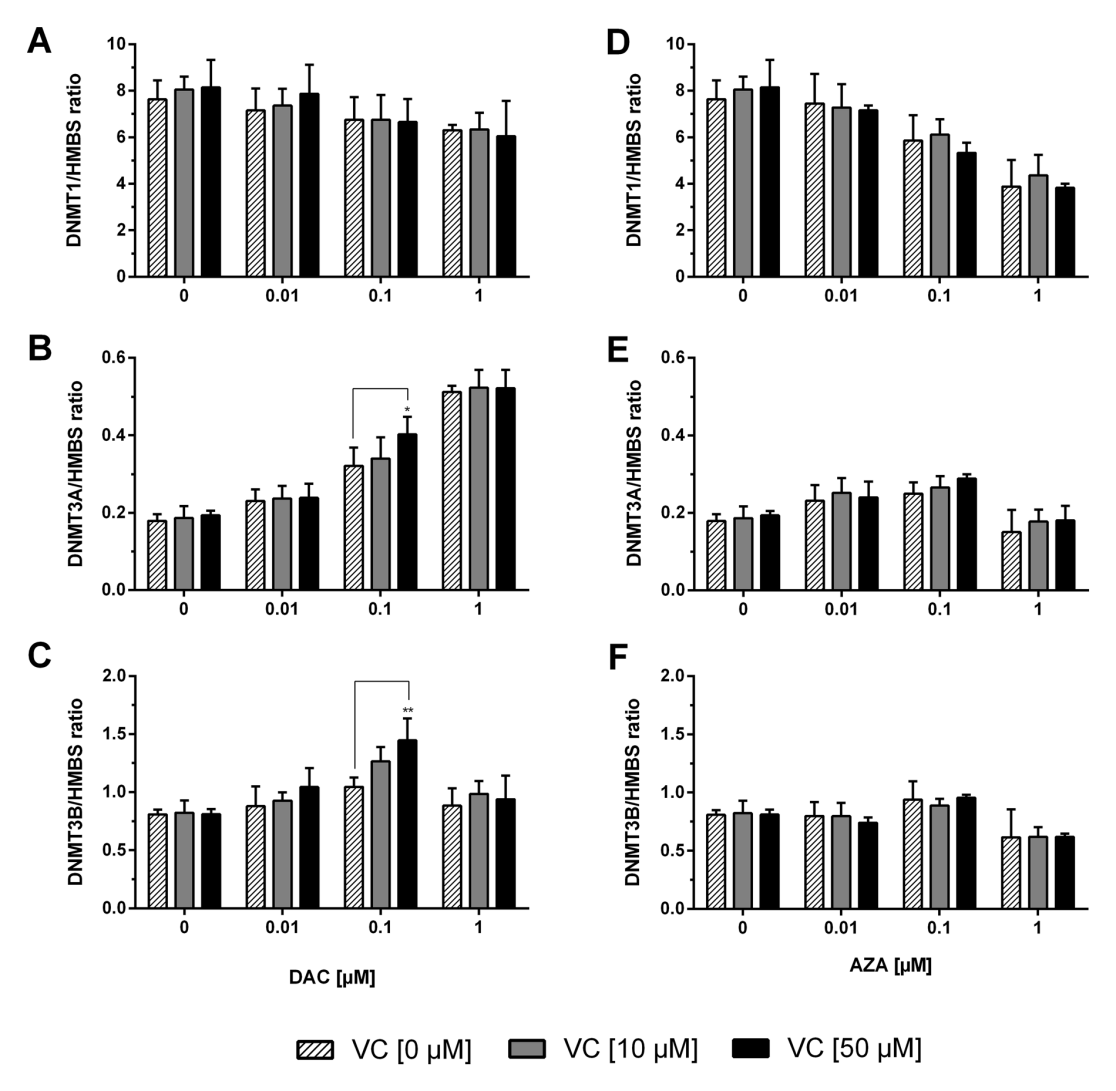
Expression profile of *DNMTs* in human colon cancer cells The mRNA levels of the *DNMTs* were determined after 72 h treatment with increasing concentrations of DAC **(A, B, C)**, AZA **(D, E, F)** in combination with or without vitamin C (0, 10 and 50 μM). The relative quantification was calculated by 2^-ΔΔCT^ method. *HMBS* served as control. Statistical significance was calculated using 2-way ANOVA and Tukey post-test: ^*^p < 0.05, ^**^p < 0.01. Error bars = SD; n=3.

To further elucidate the mechanism by which vitamin C and DAC or AZA change 5-hmdC levels, we measured the gene expressions of *DNMT1, 3A* and *3B* (Figure 5) in treated HCT116 cells. These genes are responsible for the catalysis of cytosine methylation in CG dinucleotides.

While application of DAC alone did result in decreased expression of *DNMT1,* and a significant DAC concentration-dependent effect was measured (p < 0.01), addition of vitamin C did not further alter *DNMT1* expression (Figure 5A). DAC application also significantly affected *DNMT3A* expression (p < 0.0001) causing increases of up to 2.9 fold. Notably, the effect at 0.1 μM DAC was measurably enhanced upon co-treatment with vitamin C at 10 or 50 μM (Tukey: p < 0.05) (Figure 5B). Similarly, although treatment with vitamin C or DAC alone caused no significant change to *DNMT3B* expression (Figure 5C), increases of up to 1.8 fold were seen when applied together, and which positively correlated to the concentration of both compounds below 1 μM DAC (p < 0.0001).

Although stimulation of HCT116 cells with increasing concentrations of AZA significantly modulated the expressions of *DNMT1*, *DNMT3A* and *DNMT3B* in concentration-dependent manners (p < 0.0001 in all three instances), no significant effect was measured upon co-treatment with either 10 or 50 μM vitamin C (Figure 5D-5F). A marked decrease in the expression of the three genes can be seen at 1 μM AZA; the large decrease in cell viability seen at this same concentration is notable in this regard.

**Figure 6:**
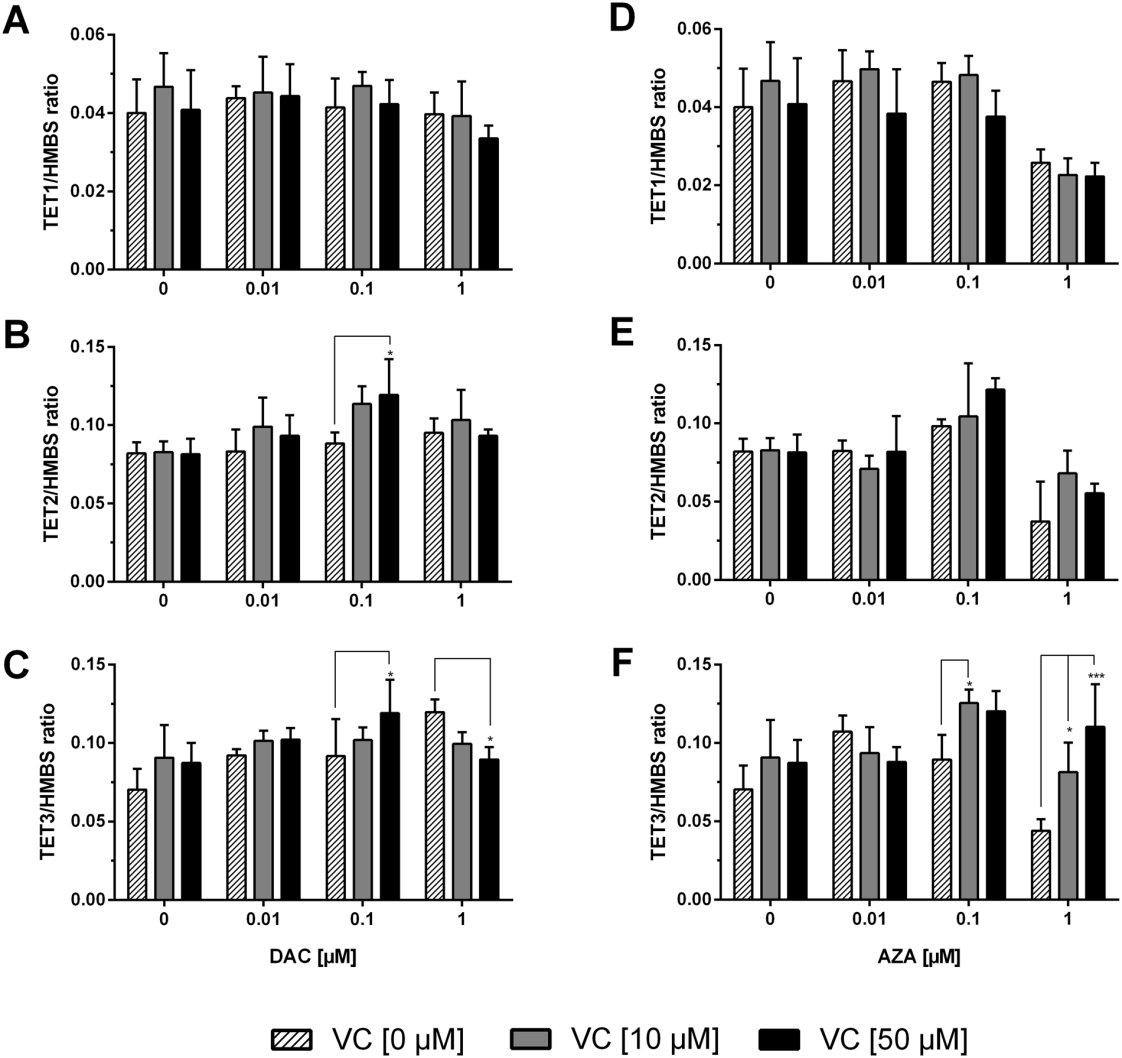
Expression profile of *TET* dioxygenases in human colon cancer cells The mRNA levels of *TETs* were determined after 72 h treatment with increasing concentrations of DAC **(A, B, C)**, AZA **(D, E, F)** in combination with or without vitamin C (0, 10 and 50 μM). The relative quantification was calculated by 2^-ΔΔCT^ method. *HMBS* served as control. Statistical significance was calculated using 2-way ANOVA and Tukey post-test: ^*^p < 0.05, ^**^p < 0.01, ^***^ < 0.001. Error bars = SD; n=3.

The expression of *TET1, 2,* and *3* – the dioxygenases responsible for the oxidation of 5-mdCs to 5-hmdC and further oxidised forms – were also assessed (Figure 6). *TET1* expression was unaffected by either DAC or vitamin C alone or in combination (Figure 6A). Although DAC and vitamin C had no significant impact on *TET2* applied alone, measurable increases were seen in combination, reaching as high as a 1.5 fold increase on the untreated cells, and a significant difference detected with 0.1 μM DAC and 50 μM vitamin C relative to matched DAC only treated cells (Tukey: p < 0.05) (Figure 6B). *TET3* expression was increased in a DAC dependent manner (p < 0.01) that was enhanced by the co-administration of vitamin C, most pronouncedly at 0.1 μM DAC, where a 1.7 fold increase was seen relative to untreated cells (Figure 6C). Interestingly, at 1 μM DAC the addition of vitamin C induced significant decreases to *TET3* expression (Tukey: p < 0.05 with 50 μM vitamin C).

Upon treatment with AZA, *TET1* transcript levels were not altered until 1 μM AZA, where decreases are observed, again correlating to the large decreases in cell viability at this AZA concentration (p < 0.001) (Figure 6D). Application of vitamin C did not significantly alter these values. A significant effect between *TET2* expression and AZA concentration was also measured (p < 0.0001), and though no significant difference was measured upon vitamin C addition, the highest observed increases were seen with combinatorial treatments; 0.1 μM AZA + 50 μM vitamin C caused a 1.5 fold increase relative to untreated cells. *TET3* expression with 0.1 μM AZA was, by contrast, significantly enhanced in the presence of 10 μM vitamin C (Tukey: p < 0.05) causing a 1.7 fold increase relative to untreated cells (Figure 6F). Interestingly, the co-administration of vitamin C was able to mitigate the decreased *TET3* expression caused by treatment with 1 μM AZA significantly (Tukey: p < 0.05 and p < 0.001 for 10 and 50 μM vitamin C respectively).

### Expression of vitamin C transporters

**Figure 7:**
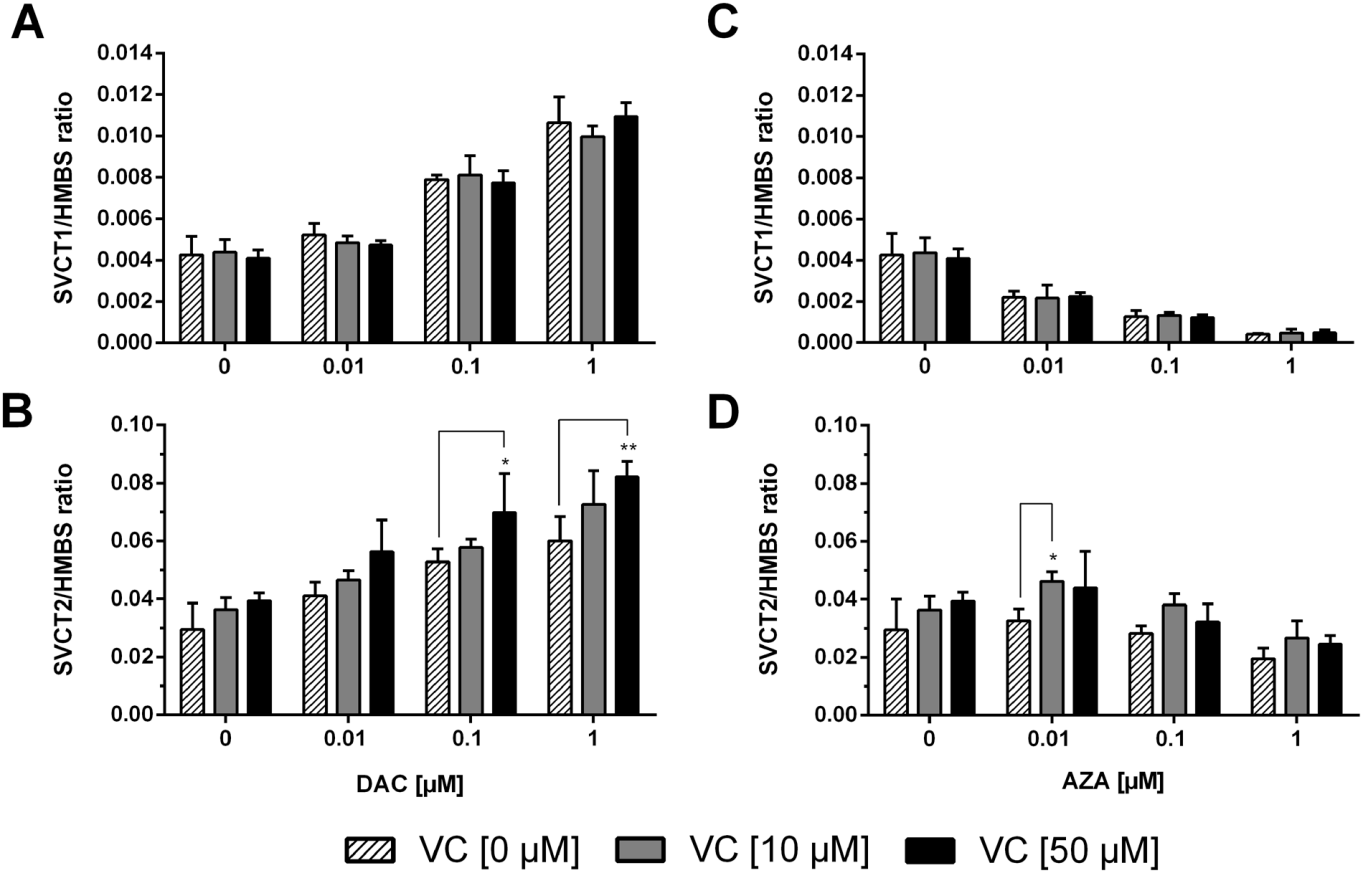
Gene expression profile of sodium dependent vitamin C transporters (*SVCT1* and *SVCT2*) in human colon cancer cells After 72 h incubation with 0.01, 0.1, or 1 μM DAC **(A, B)** or AZA **(C, D)**, alone or in combination with vitamin C (0 and 50 μM), mRNA expression levels of *SVCT1* (A, C) and *SVCT2* (B, D) in HCT116 cells were calculated, relative to the *HMBS* housekeeper gene, using the 2^ΔΔCT^ method. Statistical significance was calculated using 2-way ANOVA and Tukey post-test: ^*^p < 0.05, ^**^p < 0.01. Error bars = SD; n=3.

The cellular uptake of vitamin C is primarily facilitated by the sodium-dependent transporters SVCT1 and SVCT2. Expression changes to the transporters were analysed as one of the mechanisms by which vitamin C could exhibit its epigenetic action in combination with DAC or AZA. Therefore, it was of great interest to determine the expression levels of these *SVCTs* in cancer cells before and after exposure to DAC, AZA and vitamin C (VC). Both *SVCT1* and *SVCT2* gene transcripts were detectable in HCT116 cells (Figure 7), although the expression level of *SVCT1* was much lower than that of *SVCT2*. After exposure to DAC (Figure 7A and 7B) a dose-dependent increase in *SVCT1* was observed (p < 0.0001) that vitamin C did not affect. Similar increases were seen in *SVCT2* (p < 0.0001), but which were affected by dual administration with vitamin C, and indeed were visible even in the absence of DAC (p < 0.001). Importantly, the application of 50 μM vitamin C significantly enhanced the *SVCT2* expression induced by 0.1 (Tukey: p < 0.05) and 1 μM DAC (Tukey: p < 0.01) by, respectively, 1.3 and 1.4 fold relative to matched DAC only treatment.

Contrastingly, treatment with increasing concentrations of AZA led to a significant dose-dependent decrease of *SVCT1* expression (p < 0.0001; up to 8.8 fold) that combination with vitamin C did not alter (Figure 7C). *SVCT2* expression was not notably altered by AZA treatment except at 0.01, 0.1 μM in combination with 10 or 50 μM vitamin C (Figure 7D; Tukey: p < 0.05 and p > 0.05 respectively, 1.6 fold and 1.5 fold respectively).

### Reactivation of *CDKN1A (p21)* gene and protein expression

**Figure 8:**
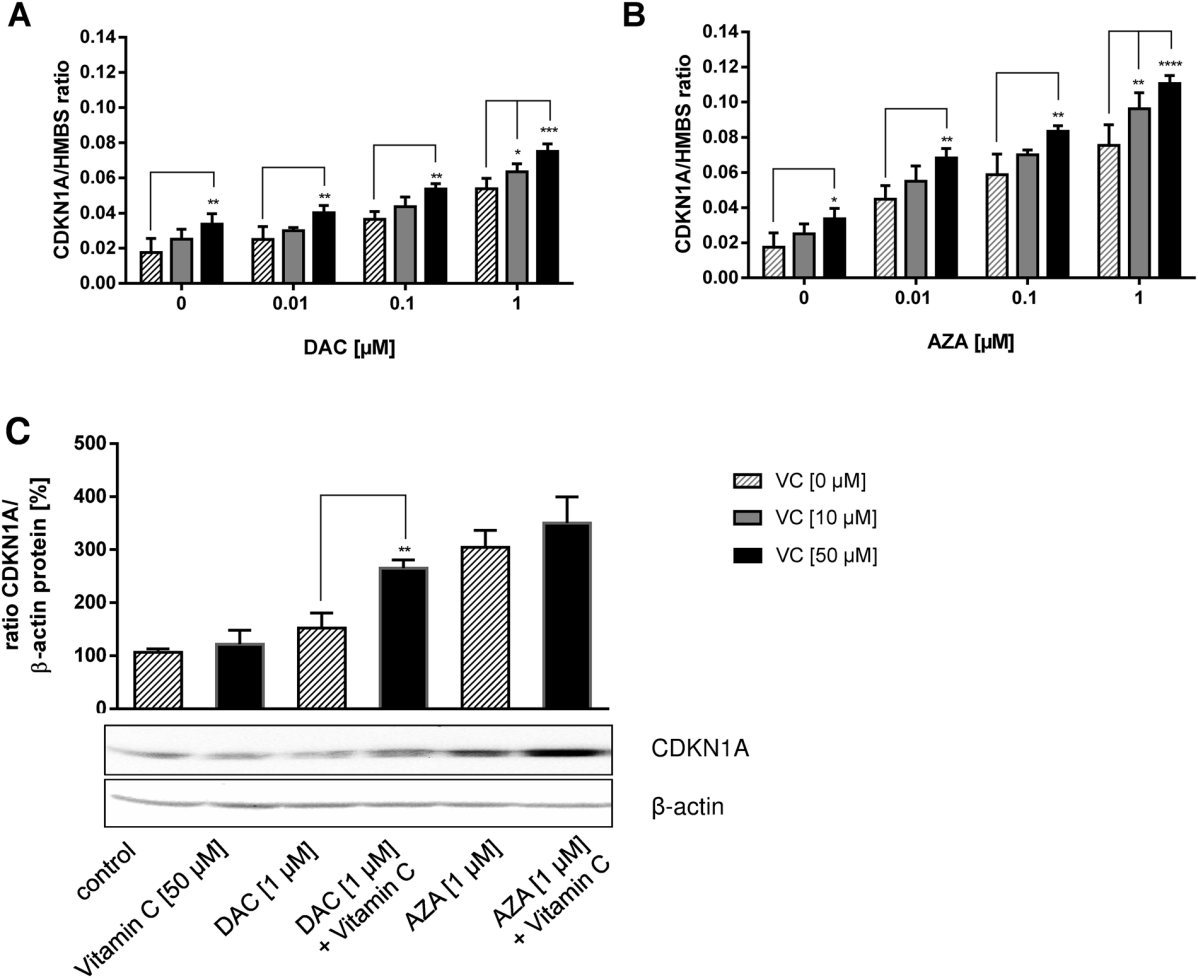
Reactivation of *CDKN1A (p21)* tumour suppressor on mRNA and protein level by DAC, AZA and vitamin C in colon cancer cells HCT116 cells were incubated with increasing concentrations of DAC or AZA, alone or in combination with 0 μM (striped bars), 10 μM (grey bars) and 50 μM (black bars) vitamin C for 72 h. The mRNA levels of *CDKN1A (p21)* after treatment with DAC **(A)** and AZA **(B)** were then calculated by 2^-ΔΔCT^ method in relation to *HMBS*, which served as a control. Additionally, the protein levels of CDKN1A (p21) were determined by Western Blotting and ECL detection **(C)**. The blotting intensities were normalized to β-actin and the untreated control. The experiments were conducted in triplicate. Statistical significance of the treated groups to the untreated control was calculated using 2-way ANOVA and Tukey post-test: ^*^p < 0.05, ^**^p < 0.01, error bars = SD; n=3.

To explore the mechanisms of DAC and AZA dependent reactivation of tumour suppressor expression in colon cancer cells, alone and in combination with vitamin C, the expression of *CDKN1A (p21)* was assessed by real-time quantitative reverse transcriptase (RT)-PCR following 72 h incubations (Figure 8A, 8B). Evidence suggests that *CDKN1A (p21)* is an important regulator of the cell cycle that is frequently silenced by epigenetic mechanisms in cancer cells [57]. In untreated HCT116 cancer cells, significant reductions in the expressions of *CDKN1A* were found, as compared to the normal colon cells (HCEC; data not shown).

Clear dose-dependent increases in *CDKN1A* expression were achieved by treatment with DAC or vitamin C alone that were significantly enhanced by combinatorial treatment (Figure 8A). These same trends were also seen on treatment with AZA in the presence or absence of vitamin C (Figure 8B). In comparison to DAC, AZA induced stronger *CDKN1A* expression at matched concentration, either alone or in combination with vitamin C (Figure 8B).

Further, we examined whether the induction of *CDKN1A* gene expression resulted in increased CDKN1A protein levels, as well. The protein expression of CDKN1A in HCT116 cells treated with DAC or AZA in combination with vitamin C was analysed by Western blot (Figure 8C). Concordantly, increases in CDKN1A protein levels were seen following treatment with 1 μM DAC (p > 0.05, 1.4 fold) or AZA (p < 0.001, 2.9 fold) (Figure 8C). Though treatment with 50 μM vitamin C alone had no impact on *CDKN1A*, it measurably enhanced the effects of DAC and AZA (Tukey: p < 0.01 and p > 0.05 respectively; 1.7 fold and 1.2 fold increases relative to DAC or AZA only treated cells respectively).

### Apoptotic induction by DAC and AZA in combination with vitamin C

**Figure 9:**
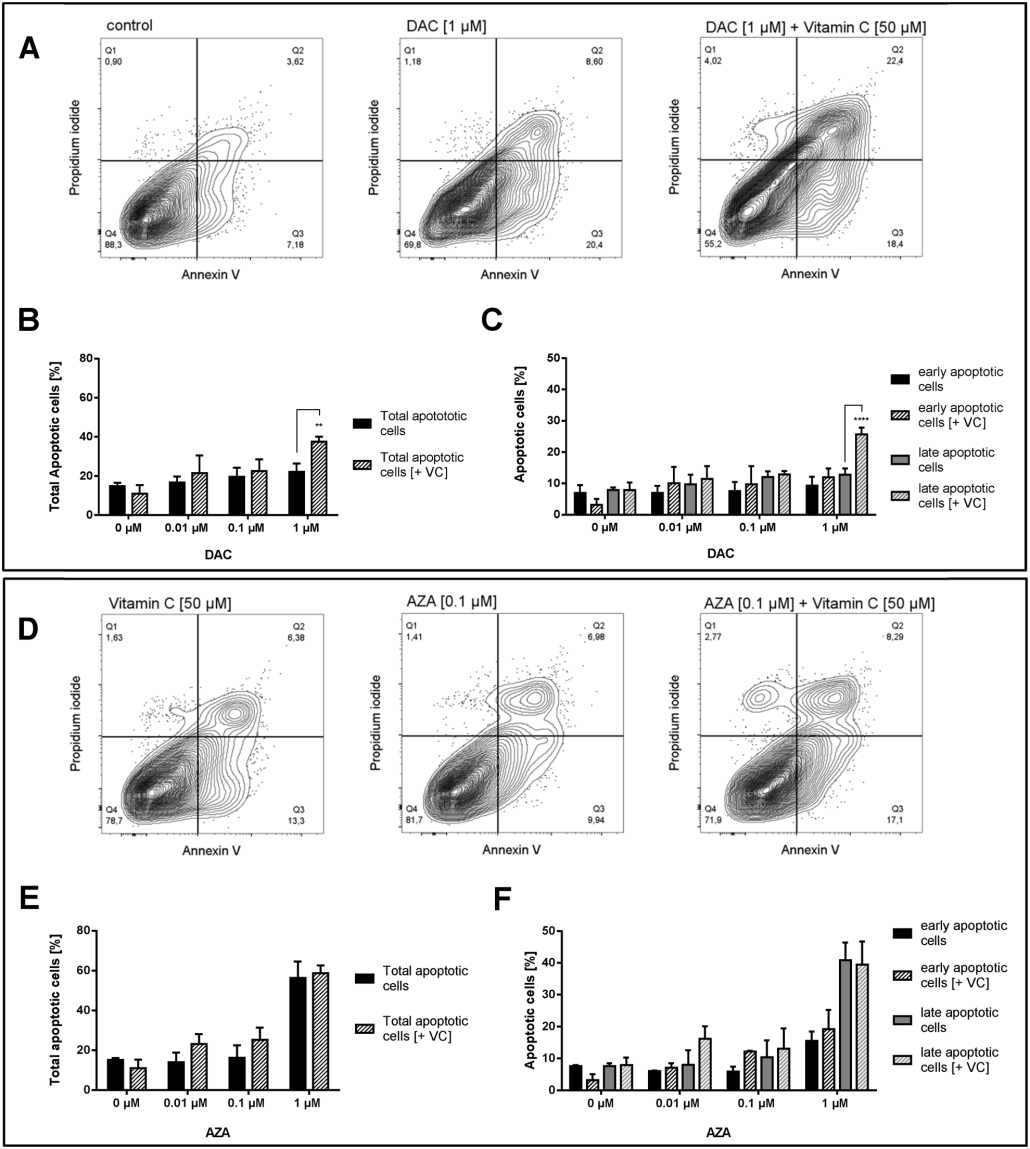
Increase of apoptotic cells in human colon cancer cells by vitamin C and DAC/AZA incubation Representative contour plots of Annexin V/propidium iodide staining in HCT116 colon cancer cells treated for 72 h with DAC **(A)** and vitamin C, alone or in combinations, as indicated. Q1 gates for necrotic cells, Q2 for late apoptosis, Q3 for early apoptosis and Q4 for healthy cells. TNFα (20 ng/mL) and cycloheximide (20 μg/mL) were used as positive controls for the induction of apoptosis. Quantitative representation of total apoptotic cells **(B)** and early + late apoptotic cells separately **(C)** after treatment with DAC with or without vitamin C, with indicated concentrations. Representative contour plots of Annexin V/propidium iodide staining in HCT116 colon cancer cells treated for 72 h with AZA in indicated concentrations and vitamin C [50 μM] **(D)**. Quantitative representation of total apoptotic cells **(E)** and early + late apoptotic cells separately **(F)** after treatment with AZA with or without vitamin C, with indicated concentrations (error bars = SD; n=3; Statistical significance of the treated groups to the untreated control was calculated using 2-way ANOVA and Tukey post-test ^****^ = p< 0.0001, ^***^ = p<0.001, ^**^ = p<0.01, ^*^ = p<0.05).

Earlier studies stated that DAC and AZA have pro-apoptotic activities in myeloid leukaemia cells [58, 59], neoplastic mast cells [60] and have the ability sensitize colorectal cancer cells to apoptosis induction by chemotherapy or immunotherapy [61, 62]. To elucidate whether the pro-apoptotic activity of DAC and AZA could be enhanced by vitamin C, combined treatments in HCT116 cells were assessed by Annexin V/propidium iodide staining and flow cytometric analysis (Figure 9). The median fluorescent intensities of cells positive for Annexin V were used to quantify apoptotic cells, of which, those positive for both Annexin V and propidium iodide were defined as late apoptotic, and those positive for Annexin V only as early apoptotic.

Following 72 h incubation with DAC, significant induction of HCT116 cell apoptosis relative to untreated cells was only seen upon treatment with 1 μM DAC and 50 μM vitamin C (Tukey: p < 0.0001; 2.5 fold increase), where the addition of vitamin C also significantly enhanced the effect of DAC alone (Tukey: p < 0.01; 1.7 fold increase) (Figure 9A and 9B). This effect appeared to derive primarily from an increase in late apoptosis, where the addition of vitamin C increased DAC induced late apoptosis 3.3 fold (Tukey: p < 0.0001) (Figure 9C). Though significant differences were also seen in early apoptosis, these derived from the apparent reduction in early apoptosis induced by vitamin C alone, and did not significantly increase the apoptosis seen in untreated cells.

Following 72 h incubation with AZA, significant dose-dependent increases in HCT116 cell apoptosis were seen (p < 0.0001), reaching up to a 3.8 fold increase relative to untreated cells (Tukey: p < 0.0001) (Figure 9D and 9E). Though slight increases in total apoptosis were seen at 0.01 and 0.1 μM AZA on addition of 50 μM vitamin C, no significant differences were detected in total apoptosis, late apoptosis or early apoptosis (Figure 9E and 9F). However, a significant increase relative to 50 μM vitamin C only treated cells was seen on co-treatment with 0.1 μM AZA (p < 0.05), though this again relates to the decrease in early apoptosis induced by vitamin C alone. Similar to DAC, it is interesting to note the emergence of a necrotic population on addition of 1 μM AZA and 50 μM vitamin C not seen with AZA alone (Figure 9D).

## DISCUSSION

Although epigenetic events are a well-studied hallmark of cancer development, very few epigenetic therapies against cancer are approved. The cytosine-analogues DAC and AZA, the most frequently used epigenetic drugs, are highly effective against haematological malignancies such as AML in older individuals [38]. Previous efforts to introduce cytosine analogues to the treatment of solid tumour malignancies have concentrated on the use of DAC and AZA in high doses in cancer cells, which led to more cytotoxic induction without epigenetic modulation [50, 51, 63, 64].

Colorectal cancer is one of the most common solid tumour cancers and epigenetic aberrations are very common [65–69]. However, as for most solid tumour cancers, an epigenetic therapy is still lacking. Therefore, we studied here the efficacy of an innovative combinatorial therapy with hypomethylating agents DAC and AZA and physiological relevant doses of vitamin C on colon cancer cells. Vitamin C has attracted attention as a potential cancer therapy through induction of oxidative stress that selectively kills cancer cells [70, 71]. However, vitamin C has also been shown to act as a potent activator and possible co-factor of the TET dioxygenases, which mediate the oxidation of 5-mdC to 5-hmdC in genomic DNA, a possible therapeutic avenue for active demethylation in aberrantly hypermethylated cancer loci [72, 73]. Notably, there is an underestimated prevalence of vitamin C deficiencies in cancer patients [74–76]. Moreover, sufficient supplementations with physiologically relevant concentrations of vitamin C have been shown to significantly improve the epigenetic outcomes of the epigenetic therapies such as DAC and AZA [74].

Here, we investigated whether vitamin C can enhance the demethylating actions of DAC or AZA, and whether such combinatorial treatments could be used as an innovative therapy in colorectal cancer. The human colon cancer cell line HCT116 was selected as an *in vitro* model of colorectal cancer. Firstly, cytotoxic assessments of DAC and AZA in HCT116 cells were performed. From these, the sub-cytotoxic concentrations of DAC (0.001–1 μM) and AZA (0.001–0.1 μM) were identified, as well as one AZA concentration of moderate toxicity (1 μM). These were selected to reflect a realistic pharmacological scenario in which efficient demethylating activity, rather than cytotoxicity, might be seen [43]. In addition, concentrations of 10 and 50 μM vitamin C were chosen to reflect plasma levels achieved by deficient and normal dietary intakes, rather than by pharmacological supplementation. Although several studies have demonstrated that vitamin C inhibits proliferation and induces apoptotic effects at very high concentrations [55, 70], our MTT viability studies confirmed our selected ascorbate doses did not induce significant toxicity to HCT116 cells. Importantly, the addition of 50 μM vitamin C did not cause further AZA or DAC induced toxicity at any of the tested concentrations.

Next, the impact of vitamin C of DAC or AZA on the state of HCT116 cell apoptosis was investigated by flow cytometry. While supplementation with physiologically relevant vitamin C concentrations did not alter the cytotoxic potential of either agent, a shift in the number of cells undergoing late apoptosis was seen, particularly for DAC treated cells where combinations with vitamin C induced the highest measured levels of apoptosis. Indeed, vitamin C resulted in significant increases to apoptotic induction with 1 μM DAC, both sub-cytotoxic concentrations alone or in combination with vitamin C. Notably, little cell necrosis was seen at even the highest AZA and DAC concentrations alone or in combination with vitamin C. These data indicate that a combination of demethylating and hydroxymethylating substances could have significant impacts on cell cycle normalization and apoptosis induction in colon cancer cells, not related to direct cytotoxic potential.

To elucidate whether epigenetic activity was increased by the combinatorial treatments of AZA or DAC with vitamin C, genome-wide changes in DNA-methylation and DNA-hydroxymethylation states were assessed by a highly sensitive LC-MS/MS approach. Previous studies have shown that global DNA methylation status is a poor marker of cell division changes within cancer cells after DAC treatment [77, 78]. This has led to the assumption that 5-hmdC/dC status is a more accurate reflection of gene re-expression for at least certain gene subsets, e.g. the tumour suppressor *CDKN1A (p21)* [79–81].

Reductions to 5-mdC levels occurred in a concentration-dependent manner for both AZA and DAC, and were not affected by the addition of vitamin C. By contrast, the demethylating agents alone mostly failed to elicit any change to 5-hmdC-levels, with only the moderately toxic dose of AZA showing any kind of effect. This is in contrast to a previous study that reported DAC could induce an increase of 5-hmdC-levels in leukaemia cell lines HL-60 (AML) and TK6 (CML) [82]. Instead, treatment with vitamin C induced concentration-dependent increases to 5-hmdC/dC levels that plateaued between 50 and 100 μM. Remarkably, combinatorial treatments with AZA or DAC resulted in significant enhancements to this effect, suggesting some form of synergism or additive effect was taking place between sub-toxic concentrations of vitamin C and AZA or DAC.

Interestingly, the combination of the moderately toxic AZA dose (1 μM) and vitamin C induced the most prominent increase in 5-hmdC levels. The results of the LC-MS/MS analysis illustrate that VC is not involved in the passive DNA hypomethylation mediated by inhibition of the DNMTs, but is involved in the active global 5-hmdC increase. Concordant gene expression data re-affirmed the role of vitamin C in AZA or DAC mediated epigenetic modulation. The expression of *ALU*, the promoter regions of which are highly CpG rich and so particularly prone to DNA methylation, were increased by AZA or DAC mediated demethylation in a concentration-dependent manner that was not affected by addition of vitamin C. Expression of *CDK1NA* was also increased in an AZA or DAC concentration-dependent manner, however here vitamin C was shown to significantly enhance this effect at the mRNA and protein level. Indeed vitamin C was capable of moderately increasing *CDK1NA* expression when applied alone. *CDKN1A* (*p21*) is a tumour suppressor that plays an important role in preventing tumour development by inhibiting cell cycle progression and inducing apoptotic signals within the cell [83]. Its increased expression may in part explain the increased prevalence of apoptosis in HCT116 cells following the combination of vitamin C to AZA or DAC treatments. However, the treatment failed to reactivate other epigenetically silenced tumour suppressors like *CDKN1B (p27)* or *CDKN2A (p16)* (data not shown).

To unravel the mechanism by which vitamin C and AZA or DAC may interact to enhance genome-wide hydroxymethylation states, the expression levels of *DNMT* and *TET* family genes were assessed. As already noted, AZA and DAC act to inhibit the actions of DNMTs, thereby preventing DNA methylation, and vitamin C enhanced TET activities resulting in increased DNA hydroxymethylation. Interestingly, while vitamin C alone had no impact on *DNMT* expression, significantly enhanced expression of *DNMT3A* and *DNMT3B* were seen when applied in combination with 0.1 μM DAC. Though the enhanced expression of DNMTs on addition of DAC is perhaps counterintuitive, this perhaps reflects a feedback loop in response to the role of DAC in depleting DNMT. Similarly, though vitamin C alone did not impact on *TET* expression, measurable enhancements to *TET2* and *TET3* expression were seen in combination with 0.1 μM AZA or DAC, an action that could well improve the hydroxymethylating activity of vitamin C. It should be noted that these effects were seen almost solely at 0.1 μM AZA and DAC, correlating only partly with hydroxymethylation states, suggesting further mechanisms are at play, including the vitamin C induced increase to the enzymatic activity of the TETs.

It was also of interest to examine the expression of the sodium-dependent transporters that bring vitamin C into the cell. Exposing HCT116 cells to 50 μM vitamin C and DAC could increase *SVCT1* and *SVCT2* expression. This led us to the hypothesis that an increase in *SVCT1* and *SVCT2* could enhance the vitamin C uptake into the cell and therefore potentiate the vitamin C related apoptosis induction in the cancer cells, possibly explaining the co-operative effect seen between vitamin C and DAC on genomic 5-hmdC/dC levels [71, 74]. In this regard, a local vitamin C deficiency, along with a decreased level of TETs, could cause loss of *SVCTs* expression that may contribute to the global loss of 5-hmdC/dC in colon adenocarcinoma cells [74]. In our study, treatment with AZA failed to induce *SVCT* expression, probably based on their different mode of action [38].

A recent study stated that the combinational therapy of elderly AML patients with DAC and vitamin C led to improvements in overall survival and remission rates compared to patients treated with DAC alone [84]. In accordance to our data, it was found that low doses of vitamin C (300 μM) could enhance the effects of 2.5 μM DAC on the leukaemia cell lines HL60 and NB4, reducing proliferation, increasing apoptosis and enhancing TET2 activity. In our study, we were able to achieve similar *in vitro* results in colorectal cancer cells with even lower concentrations of the compounds. In contrast, though increased *TET* expression was identified, this only occurred within a limited DAC concentration range with apparent decreases at lower doses than used by Zhao (2018) and co-workers [84]. However, a significant increase of 5-hmdC/dC levels was demonstrated after combinatorial treatments with vitamin C compared to AZA or DAC treatment alone. This led us to suggest that not an increased TET-expression but an increased TET activity may lead to the higher levels of 5-hmdC/dC.

The present study shows an important impact on the epigenetic landscape of colorectal cancer cells through the combined actions of functionally distinct substances, leading to significant improvements in the apoptotic induction of HCT116 cells. Furthermore, the combination of sub- or low-micromolar doses of demethylating agents like DAC and AZA with moderate, physiological levels of vitamin C was shown to result in enhanced epigenetic actions, increased expression of suppressed tumour suppressors, and a positive shift in the apoptotic state without significant cytotoxic induction. Although this is not the first time a counter-intuitive link between the application of hypomethylating agents and increased genomic hmdC levels has been identified [82], this is the first time a direct link between this and vitamin C levels has been observed. In conclusion, our results indicate epigenetic therapies mediated by sub-micromolar concentrations of DAC or AZA are capable of enhanced anti-cancerous activities when administered in combination with vitamin C at physiologically relevant doses. Vitamin C deficiencies are common among many cancer patients [76]. As such, our data suggest that therapeutic responses to low, non-toxic doses of DNMT inhibitors can be improved by additional vitamin C supplementation.

## MATERIALS AND METHODS

### Cell culture and treatment

HCT116 (ATCC no. CCL-247), a human colorectal carcinoma cell line, was purchased from the American Type Culture Collection (ATCC; https://www.atcc.org/). Cells were cultured in Dulbecco’s Modified Eagle’s Medium (DMEM) with 2 mM L-glutamine supplemented with 10% fetal bovine serum (FBS), 45 IU/ml penicillin, and 45 IU/ml streptomycin. The cell lines were confirmed as negative for mycoplasma infection within six months prior to use. HCT116 cells were treated daily with DAC or AZA (0, 10, 100 or 1000 nM) for three consecutive days, alone or in combination with vitamin C (0, 10 or 50 μM). DAC, AZA and vitamin C were administered at the same time points.

### Cell viability assay

Potential cytotoxic effects induced by vitamin C, AZA and DAC were assessed by 3-(4, 5-dimethylthiazol-2-yl)-2, 5-diphenyltetrazolium bromide (MTT) reduction assay, as previously described [85]. HCT116 cells were seeded into 96-well plates (TPP, Trasadingen, Switzerland). After 24 h, the indicated concentrations were added for 24, 48 and 72 h. Subsequently, the cells were incubated with 100 μL MTT solution (0.5 mg/mL in PBS) for 4 h. After removing the supernatants, 50 μL dimethyl sulfoxide was added to dissolve the formazan salt and its optical density (OD) was measured using a microplate reader (Tecan, Crailsheim, Germany) at 540 nm. The positive controls were treated with 0.002% SDS. A cell viability < 75% predicts cytotoxic effects.

### Determination of genome-wide DNA methylation and hydroxymethylation using isotope-dilution liquid chromatography tandem-mass spectrometry (LC-MS/MS)

Samples of genomic DNA (20 μg) were hydrolyzed to 2’-deoxynucleosides using micrococcal nuclease from *Staphylococcus aureus*, bovine spleen phosphodiesterase, and calf intestinal alkaline phosphatase (all from Sigma-Aldrich, Taufkirchen, Germany) as described previously [86] with slight modifications. 10 μL of 50 nM 5-hmdC-d_3_ (Toronto Research Chemicals, Toronto, Canada) was added to the DNA digestion mixture as an internal standard, and the incubation times of the two-step hydrolysis were 1 h each. Afterwards, DNA hydrolysates were centrifuged (5 min; 16,000 x *g*) and 10 μL of their supernatants taken for quantification of dC and 5-mdC by means of the stable isotopically labelled reference compounds [^15^N_2_,^13^C_1_]dC and 5-mdC-d_3_ (both from Toronto Research Chemicals, Toronto, Canada) as recently described [87]. The remaining DNA hydrolysates (∼ 310 μL) were evaporated to dryness under reduced pressure using a Savant SpeedVac Concentrator (Thermo Fisher Scientific, Dreieich, Germany). The dried residues were then mixed with 100 μL methanol, briefly vortexed, and stored overnight (-20°C). The next day, samples were thoroughly vortexed (1,400 rpm) for 10 min followed by centrifugation at 16,000 x *g* for 10 min. Supernatants were transferred to new sample tubes. Extraction of the protein pellets was repeated by addition of a further 100 μL methanol and vortexing (1,400 rpm) for 5 min. After centrifugation at 16,000 x *g* for 10 min, both methanolic fractions were combined and evaporated to dryness under reduced pressure. The dried residues were reconstituted in 50 μL water containing 0.0075% formic acid, ultra-sonicated for 10 min, vortexed (1,400 rpm) for 5 min, and centrifuged for 5 min at 16,000 x *g*. LC-MS/MS analyzes of the supernatants were conducted with an Agilent 1260 Infinity LC system coupled to an Agilent 6490 triple quadrupole mass spectrometer (both from Waldbronn, Germany) interfaced with an electrospray ion source operating in the positive ion mode (ESI+). Chromatographic conditions and settings of the ESI source were as described recently for quantification of dC and 5-mdC [87] with the following exceptions: a longer separation column (Agilent Poroshell 120 EC-C18, 2.7 μm, 3.0 x 150 mm) was used and the injection volume was 5 μL. Quantification of 5-hmdC in relation to its stable isotope labelled standard, both eluted at 4.9 min from the LC column (retention times of dC and 5-mdC were 4.7 and 6.0 min, respectively), was carried out using the multiple reaction monitoring (MRM) approach. The following mass transitions (loss of 2’-deoxyribose) were used as quantifiers (optimized collision energies in parentheses): 5-hmdC: *m/z* 258.1 → 142.0 (8 eV) and 5-hmdC-d_3_: *m/z* 261.1 → 145.0 (8 eV). Additional mass transitions were recorded for unambiguous identification. The dwell time for each of the four mass transitions analyzed was 50 ms.

### Apoptosis assay

Levels of apoptotic and dead cells were determined by flow cytometry using eBioscience™ Annexin V Apoptosis Detection Kit APC (Thermo Fisher, Darmstadt, Germany). Briefly, 2 x 10^5^ HCT116 cells/well were seeded into 6-well plates (TPP, Trasadingen, Switzerland). After 24 h, the cells were incubated with the substances at the indicated concentrations for 72 h. Incubation with TNFα (20 ng/mL) and cycloheximide (20 μg/mL) for 72 h served as positive control for apoptosis induction. Subsequently, the cells were washed and stained with Annexin V antibody and propidium iodide (PI) according to the manufacturer’s instructions. The cells were distinguished between viable cells (Annexin V− / PI−), early apoptotic cells (Annexin V+ / PI−), late apoptotic/necrotic cells (Annexin V+ / PI+) and late necrotic cells (Annexin V− / PI+). Per run 10,000 events were counted and analyzed on a FACSCanto II (BD Biosciences, Heidelberg, Germany). FlowJo software (Treestar, Ashland, USA) was used for data analysis.

### RNA extraction and quantitative real-time PCR

RNA extraction was performed with the RNA High Pure RNA Kit (Roche, Mannheim, Germany) according to the manufacturer’s instructions. The cDNA synthesis was then conducted using the RevertAid reverse transcriptase (Thermo Fisher, Darmstadt, Germany) and 0.5-5 μg (ideally 3 μg) RNA. The qRT-PCR was performed using the Maxima SYBR Green qPCR Mix (ThermoFisher, Darmstadt, Germany) on a LightCycler 480 II Real-Time PCR system (Roche, Mannheim, Germany). Quantification was performed using the ΔΔ Ct method with *hHMBS* expression as an internal reference. Melt curve analysis confirmed that all the qRT-PCR products were generated in the form of double-stranded DNA. The primers used are listed in Table 1.

**Table 1:** Primer sequences and fragment sizes for qPCR experiments

Target gene	Sequence	Fragment size (bp)
*hHMBS*	fw: ACCAAGGAGCTTGAACATGC rv: GAAAGACAACAGCATCATGAG	143
*hDNMT1*	fw: ACCTGGCTAAAGTCAAATCC rv: ATTCACTTCCCGGTTGTAAG	80
*hDNMT3a*	fw: ACTACATCAGCAAGCGCAAG rv: CATCCACCAAGACACAATGC	359
*hDNMT3b*	fw: CCAGCTCTTACCTTACCATC rv: CAGACATAGCCTGTCGCTTG	285
*hTET1*	fw: GCTGCTGTCAGGGAAATCAT rv: ACCATCACAGCAGTTGGACA	209
*hTET2*	fw: CCAATAGGACATGATCCAGG rv: TCTGGATGAGCTCTCTCAGG	232
*hTET3*	fw: TCGGAGACACCCTCTACCAG rv: CTTGCAGCCGTTGAAGTACA	179
*CDKN2A (p16)*	fw: GAGCAGCATGGAGCCTTC rv: CCTCCGACCGTAACTATTCG	124
*CDKN1A (p21)*	fw: AGTGGACAGCGAGCAGCTGA rv: TAGAAATCTGTCATGCTGGTCTG	381
*CDKN1B (p27)*	fw: AAACGTGCGAGTGTCTAACGGGA rv: CGCTTCCTTATTCCTGCGCATTG-	456
*SVCT1*	fw: TCATCCTCCTCTCCCAGTACCT rv: AGAGCAGCCACACGGTCAT	141
*SVCT2*	fw: TCTTTGTGCTTGGATTTTCGAT rv: ACGTTCAACACTTGATCGATTC	106

### Protein extraction and western blot

Briefly, cell pellets were lysed in 200 μL RIPA buffer (50 mM Tris/HCl, 150 mM NaCl, 1% Nonidet P-40, 0.5% sodium deoxycholate, 0.1% SDS in PBS) and incubated for 30 min at 4 °C. Subsequently, cell lysates were centrifuged at 10,000 x *g* for 30 min, and the supernatant used for protein analysis.

Western blotting was performed according to standard procedure. Protein samples (50 μg per lane) were separated by SDS-PAGE, and then transferred to PVDF membrane. Following blocking (5% milk powder in TBST buffer) membranes were probed with antibodies against CDKN1A (Abcam, Cambridgeshire, UK) and anti-β-actin (Abcam, Cambridgeshire, UK), followed by reaction with the corresponding horseradish peroxidase-conjugated secondary antibody (Cell Signaling, Boston, USA). Proteins were visualized using the ECL detection system (Thermo Scientific) and ChemiDoc™ system (Bio-Rad Laboratories, Munich, Germany).

### Statistical analysis

Statistically significant differences were determined two-way ANOVA followed Tukey post hoc test, where p-values ≤ 0.05 were considered as significant. Differences in apoptosis induction were calculated by 2 way ANOVA analysis, where p-values ≤ 0.05 were considered as significant (^*^ = p < 0.05, ^**^ = p < 0.01, ^***^ = p < 0.001, ^****^ = P < 0.0001). All statistical analyses were conducted using the software GraphPad Prism (Graphpad Software, Inc., La Jolla, USA).

## Abbreviations

5-mdC: 5-methyl-2’-deoxycytidine
5-hmdC: 5-hydroxymethyl-2’-deoxycytidine
5-fdC: 5-formyl-2’-deoxycytidine
5-cadC: 5-carboxy-2’-deoxycytidine
ALU: athrobacter luteus
AML: acute myeloid leukemia
APC: adenomatous polyposis coli
ATCC: American type culture collection
AZA: 5-aza-cytidine
CDKN1A: cyclin-dependent kinase inhibitor 1
CDKN2A: cyclin-dependent kinase inhibitor 2A
CDKN2B: cyclin-dependent kinase inhibitor 2B
DAC: 5-aza-2’-deoxycytidine
DNMTs: DNA methyltransferases
FDA: US Food and Drug Administration
HMBS: hydroxymethylbilane synthase
ITGA4: integrin a subunit 4
LC-MS/MS: liquid chromatography tandem-mass spectrometry
MTT: 3-(4, 5-dimethylthiazol-2-yl)-2, 5-diphenyltetrazolium bromide
P4H: prolyl-4-hydroxylase
SVCT1: sodium dependent vitamin C transporter 1
SVCT2: sodium dependent vitamin C transporter 2
TET: ten eleven translocation
TDG: thymine-DNA glycosylase
TFPI2: tissue factor pathway inhibitor 2
VC: vitamin C
Wnt: wingless/Int1
